# Analysis of HCV quasispecies dynamic under selective pressure of combined therapy

**DOI:** 10.1186/1471-2334-13-61

**Published:** 2013-02-01

**Authors:** Ana CG Jardim, Cíntia Bittar, Renata PA Matos, Lílian HT Yamasaki, Rafael A Silva, João RR Pinho, Roberta M Fachini, Claudia MA Carareto, Isabel MVG de Carvalho-Mello, Paula Rahal

**Affiliations:** 1 Departament of Biology, Institute of Bioscience, Language and Exact Science, São Paulo State University, São José do Rio Preto, SP, Brazil; 2 Division of Gastroenterology Laboratory of Applied Molecular Hepatology, Hepatitis Section, Federal University of São Paulo, São Paulo, SP, Brazil; 3 Departament of Gastroenterology, São Paulo Institute of Tropical Medicine, School of Medicine, University of São Paulo, São Paulo, SP, Brazil; 4 Department Hepatology, São José do Rio Preto School of Medicine, São Paulo, SP, Brazil

## Abstract

**Background:**

The quasispecies composition of Hepatitis C virus (HCV) could have important implications with regard to viral persistence and response to interferon-based therapy. The complete NS5A was analyzed to evaluate whether the composition of NS5A quasispecies of HCV 1a/1b is related to responsiveness to combined interferon pegylated (PEG-IFN) and ribavirin therapy.

**Methods:**

Viral RNA was isolated from serum samples collected before, during and after treatment from virological sustained responder (SVR), non-responder (NR) and the end-of-treatment responder patients (ETR). NS5A region was amplified, cloned and sequenced. Six hundred and ninety full-length NS5A sequences were analyzed.

**Results:**

This study provides evidence that lower nucleotide diversity of the NS5A region pre-therapy is associated with viral clearance. Analysis of samples of NRs and the ETRs time points showed that genetic diversity of populations tend to decrease over time. Post-therapy population of ETRs presented higher genetic distance from baseline probably due to the bottleneck phenomenon observed for those patients in the end of treatment. The viral effective population of those patients also showed a strong decrease after therapy. Otherwise, NRs demonstrated a continuous variation or stability of effective populations and genetic diversity over time that did not seem to be related to therapy. Phylogenetic relationships concerning complete NS5A sequences obtained from patients did not demonstrate clustering associated with specific response patterns. However, distinctive clustering of pre/post-therapy sequences was observed. In addition, the evolution of quasispecies over time was subjected to purifying or relaxed purifying selection. Codons 157 (P03), 182 and 440 (P42), 62 and 404 (P44) were found to be under positive selective pressure but it failed to be related to the therapy.

**Conclusion:**

These results confirm the hypothesis that a relationship exists between NS5A heterogeneity and response to therapy in patients infected with chronic hepatitis C.

## Background

Hepatitis C virus (HCV) is the major etiological agent of chronic hepatitis worldwide [1]. Chronic infection can progress to liver cirrhosis with risk for the development of hepatocellular carcinoma [2,3].

The current treatment for chronic hepatitis C is based on interferon (IFN) or pegylated IFN in combination with ribavirin (RBV), leading to a sustained virological response in approximately 50% of patients infected with genotypes 1a/1b [4,5]. Several host parameters, disease characteristics and virus-related factors are relevant to the possibility of viral clearance after therapy [6,7].

A member of the family *Flaviviridae*, HCV is an enveloped virus with a positive, single-stranded RNA genome approximately 9.5 kb in length, encoding a single polyprotein of approximately 3000 amino acids that is co- and post-translationally cleaved by viral and cellular proteases into structural and non structural proteins [8].

HCV is classified into seven genotypes, HCV-1 to −7, with each genotype being further subdivided into subtypes such as HCV-1a and 1b [9,10]. Furthermore, in infected individuals, HCV circulates as a population of several closely related viral variants referred to as “quasispecies” [11,12]. New variants are continuously generated during viral replication as a result of errors made by the viral RNA-dependent RNA polymerase, which lacks proofreading activity, during rapid replication [13]. The quasispecies nature of HCV could have important implications for viral persistence, pathogenicity and resistance to anti-viral agents [14,15].

The non-structural 5A (NS5A) protein is implicated in interferon resistance [16]. Enomoto et al. [17,18] suggested that the genetic heterogeneity of a specific domain of the NS5A region of HCV, termed the IFN sensitivity determining region (ISDR), was related to treatment responses in Japanese patients with HCV genotype 1b infection. This is a controversial issue, but analysis of published information supports the hypothesis that a relationship exists between NS5A heterogeneity and response to therapy [19-22].

NS5A is an RNA binding phosphorylated protein comprising three domains separated by trypsin-sensitive low complexity sequences (LCS I and LCS II) and an N-terminal amphipathic alpha-helix that anchors the protein to intracellular membranes [23-25]. Full-length NS5A protein appears to be located exclusively in the cytoplasm. However, N-terminal deleted forms have been found in the nucleus, suggesting that they can move to the nucleus and act as potent transcriptional activators [26-28].

The NS5A region has generated much interest owing to its association with responses to therapy. In the present study, the evolution of the complete NS5A region of HCV genotypes 1a and 1b was examined in patients undergoing pegylated IFN plus ribavirin therapy. The results demonstrate that higher diversity and complexity of quasispecies are related to lower rates of response to peginterferon and ribavirin therapy. Therefore, quasispecies variability of the NS5A region could help understand the mechanism underlying treatment failure in patients infected with chronic hepatitis C.

## Results

### Patient characteristics

The characteristics of the patients are presented in Table 1. The population was predominantly male (NR 75% vs. SVR 67% vs. ETR 100%); 55% of patients were infected with HCV genotype 1a (NR 25% vs. SVR 67% vs. ETR 75%) and 45% of patients were infected with HCV genotype 1b (NR 75% vs. SVR 33% vs. ETR 25%). The mean age of the patients was 43 ± 13.9 years (NR 46.3 ±10.6 vs. SVR 39.7 ± 2.6 vs. 42.3 ± 6.2). No statistically significant differences were observed between groups.

**Table 1:** Clinical and virological characteristics of patients

**Patient and type of treatment response**^**a**^	**Sex**	**Age**	**HCV Genotype**	**Sample**	**Viral Load log UI/ml**	**Genetic Distance**	**Genetic Distance from baseline**^**b**^	**Shannon Entropy**
**SVR**								
P05	F	44	1b	●	6.04	0.006 ± 0.001		0.0096
P35	M	40	1a	●	6.25	0.005 ± 0.001		0.0074
P40	M	35	1a	●	6.57	0.004 ± 0.001		0.0042
**NR**								
P08	F	41	1a	●	5.80	0.008 ± 0.001		0.0126
				12wt	4.59	0.009 ± 0.001	0.009 ± 0.001	0.0113
				14d	6.18	0.011 ± 0.001	0.014 ± 0.002	0.0157
				2m	6.46	0.008 ± 0.001	0.014 ± 0.002	0.0103
				6m	6.40	0.007 ± 0.001	0.015 ± 0.002	0.0156
P11	M	72	1b	●	6.85	0.017 ± 0.001		0.0235
				12wt	3.71	0.007 ± 0.001	0.020 ± 0.003	0.0140
				14d	6.27	0.005 ± 0.001	0.032 ± 0.004	0.0134
				2m	6.73	0.007 ± 0.001	0.034 ± 0.004	0.0132
				6m	>7.2	0.012 ± 0.002	0.030 ± 0.004	0.0192
P146	M	21	1a	●	6.92	0.012 ± 0.002		0.0202
				12wt	5.54	0.006 ± 0.001	0.014 ± 0.002	0.0097
				14d	6.43	0.012 ± 0.001	0.020 ± 0.002	0.0172
				2m	6.76	0.012 ± 0.001	0.020 ± 0.002	0.0177
				6m	6.96	0.008 ± 0.001	0.017 ± 0.002	0.0119
P44	M	51	1b	●	6.50	0.016 ± 0.002		0.0139
				12wt	6.13	0.019 ± 0.002	0.019 ± 0.002	0.0311
				14d	6.24	0.014 ± 0.001	0.023 ± 0.003	0.0206
				2m	7.18	0.014 ± 0.001	0.023 ± 0.003	0.0216
				6m	6.83	0.014 ± 0.001	0.024 ± 0.003	0.0216
**ETR**								
P03	M	39	1a	●	5.95	0.011 ± 0.001		0.0131
				28d	4.99	0.004 ± 0.001	0.041 ± 0.005	0.0094
				2m	5.39	0.003 ± 0.001	0.039 ± 0.005	0.0058
				3m	4.74	0.003 ± 0.001	0.039 ± 0.005	0.0055
				4m	5.03	0.002 ± 0.001	0.039 ± 0.005	0.0073
				5m	5.26	0.001 ± 0.001	0.038 ± 0.005	0.0033
				6m	5.08	0.012 ± 0.000	0.038 ± 0.005	0.0038
P37	M	27	1a	●	6.00	0.005 ± 0.001		0.0137
				2m	6.63	0.001 ± 0.001	0.017 ± 0.003	0.0055
				3m	6.83	0.002 ± 0.001	0.016 ± 0.003	0.0038
				4m	6.30	0.003 ± 0.001	0.016 ± 0.003	0.0066
				5m	6.10	0.009 ± 0.001	0.017 ± 0.003	0.0074
				6m	6.58	0.014 ± 0.001	0.019 ± 0.003	0.0109
P42	M	56	1b	●	6.66	0.016 ± 0.002		0.0161
				4m	6.24	0.014 ± 0.002	0.030 ± 0.003	0.0162
				5m	6.28	0.017 ± 0.002	0.028 ± 0.003	0.0141
				6m	6.34	0.022 ± 0.002	0.030 ± 0.003	0.0145
P47	M	47	1a	●	6.05	0.033 ± 0.002		0.0268
				2m	5.67	0.027 ± 0.003	0.039 ± 0.004	0.0466
				3m	5.92	0.003 ± 0.002	0.039 ± 0.004	0.0391
				4m	5.57	0.011 ± 0.001	0.037 ± 0.004	0.0072
				5m	5.87	0.009 ± 0.002	0.038 ± 0.004	0.0197
				6m	2.93	0.011 ± 0.002	0.041 ± 0.004	0.0171

^a^SVR, sustained virological response; NR, non-response; ETR, end-of-treatment response.

●=before treatment; wt=weeks of treatment; d=days after the end of treatment; m=months after the end of treatment.

The levels of viremia in baseline samples did not differ significantly among the patient groups (NR 6.52 vs. SVR 6.39 vs. ETR 6.17 log IU/ml). Patients who did not respond to therapy had a significant decrease in viremia after 12 weeks of treatment (4.99 ± 0.94 log IU/ml; *p=0.002*), with recovering levels in samples collected after therapy was complete (6.30 – 6.86 log IU/ml). The viral load values varied among the samples from end of treatment responders. The mean viremia levels for these patients ranged from 4.99 to 5.90 log IU/ml (Table 1).

### Nonsense mutations in the NS5A region are observed in vivo

**Figure 1:**
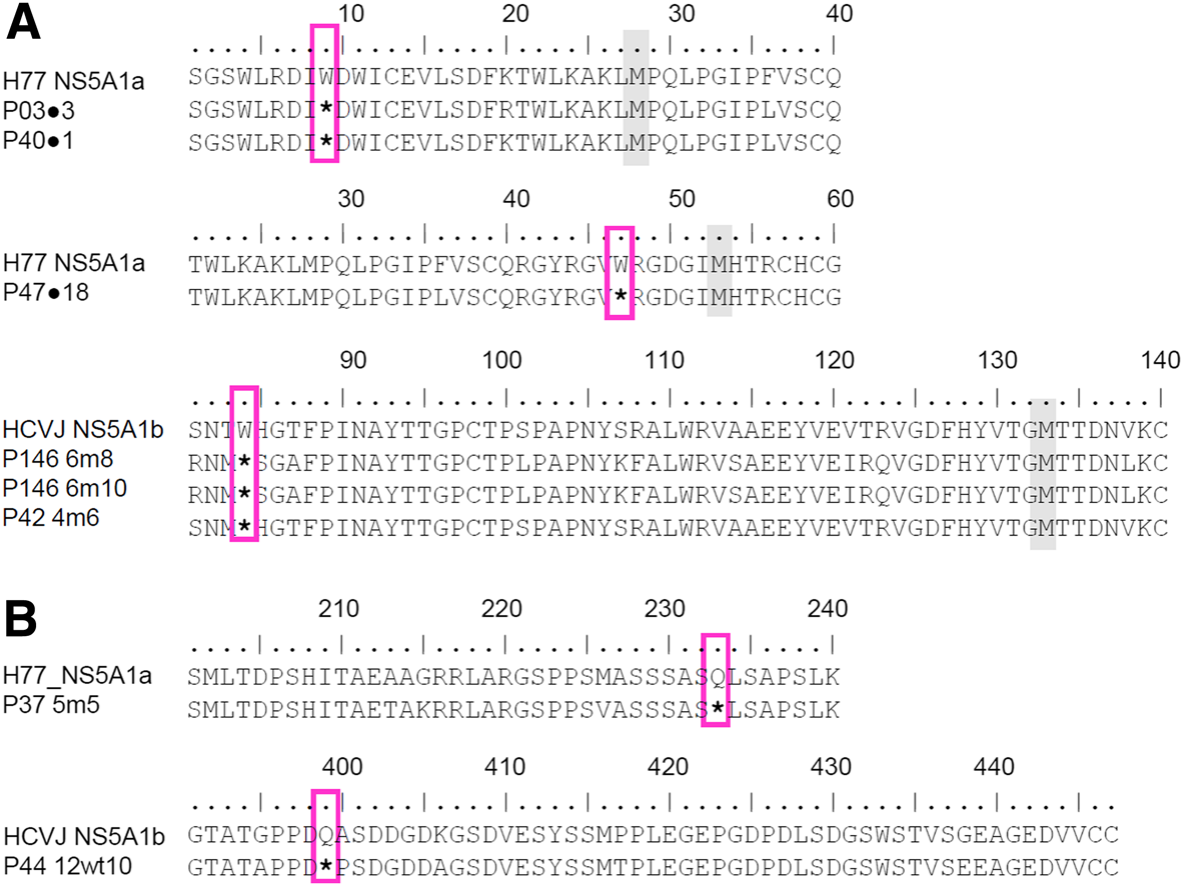
**Alignment of sequence representation demonstrating the nonsense mutations observed in this study. A**) Nonsense mutation at amino-terminal of the NS5A genomic region, positions 9, 47 and 84. **B**) One nonsense mutation located at the middle of the NS5A genomic region position 233, and one nonsense mutation located at the carboxy-terminal of the NS5A genomic region, position 399. Pink boxes indicate nonsense mutations. The next methionines are highlighted in gray. References sequences for genotypes 1a (H77_NS5A1a) and 1b (HCVJ_NS5A1b) are indicated.

Eight nonsense mutations were detected in the NS5A region after analysis of the 690 sequences dataset, two of them being common to more than one sequence. Codon 9 nonsense mutation was observed in two clones (before treatment samples from patients P03 and P40) and codon 84 in three clones (two clones in the P146 6m and one clone in the P42 4m sample). Nonsense mutations were also observed in codons 47 (before treatment sample from P47), 233 (P37 5m) and 399 (P44 12wt) (Figure 1). The majority of nonsense mutations were detected in Domain I of the NS5A region (codons 47 and 84), with only one being located in domain III (codon 399), one in the Alpha Helix (codon 9) region and one in the Low Complexity Sequence I Region (codon 233). The numeration of codons was based on the NS5A protein, the first codon being the first amino acid coded for in the NS5A genomic region.

Part of this nonsense mutation could provide a functional NS5A protein. Therefore, the sequences at the N-terminal region of NS5A in which the nonsense mutation occurred were analyzed to locate the next methionine residue. The methionines are highlighted in Figure 1A. The nonsense mutation 399 was not investigated owing to the presence of a methionine after it. This sequence could provide functional proteins despite missing a C-terminal portion (Figure 1B).

### Nucleotide diversity of NS5A and sustained response to therapy

**Figure 2:**
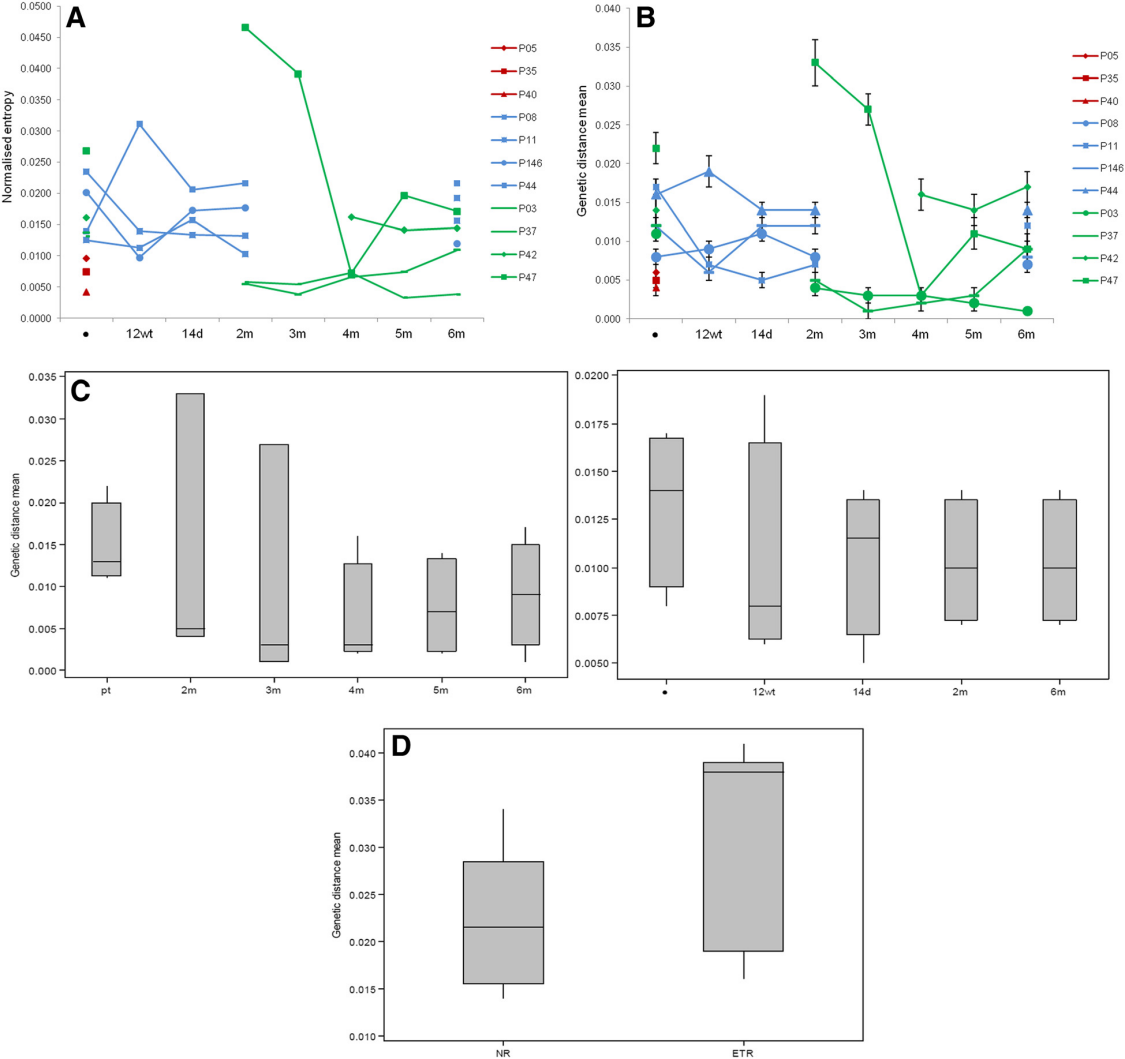
**Genetic variability of HCV quasispecies in samples collected over time. A**) Genetic complexity calculated using normalized entropy (Shannon entropy). **B**) Diversity of HCV quasispecies calculated using genetic distance (p-distance). Patterns of response to therapy are represented by colours: sustained virological responders in red, non-responders in blue and end-of-treatment responders in green. **C**) Genetic diversity by the mean of genetic distance of each time-point collected. On the left: the end-of-treatment-responders. On the right: non-responders. **D**) Genetic diversity by the mean of genetic distance between every sample and baseline. Comparisons between non-responders and end-of-treatment responders. P=value calculated using Fisher’s test or paired *t*-test.

Shannon entropy analysis showed that the genetic diversity of the NS5A region at baseline was significantly lower for the SVR group (mean value 0.00706) than for the NR (0.017529) and ETR groups (0.017427) (*p=0.0253* and *p=0.0265*, respectively) (Figure 2A). The genetic distance was also significantly lower for the SVR group (0.0050) than for the NR (0.0133) and ETR (0.0148) groups (*p=0.021* and *p=0.022*, respectively; Figure 2B). These results demonstrated that quasispecies of SVR samples at baseline are less diverse than those of NR and ETR samples.

The mean entropy and genetic distance of during-treatment samples for NR and after the completion of therapy for NR and ETR samples were comparable when the results of each patient were analyzed individually. Generally, values had a propensity to decrease in samples collected during and after the end of treatment, but no profile was detected for patients in the same response group (Table 1 and Figure 2). However, significant differences were observed when each group was compared by the mean genetic distance of two time-points. For NRs, 6m post-therapy variants showed significant lower diversity than baseline quasispecies (baseline: 0.01325 *vs* 6m: 0.01025; *p=0.046*). ETRs showed values close to significance for mean genetic distance but differences were significant when Shannon entropy was analyzed between pre and 5m post-therapy (mean genetic distance - baseline: 0.01475 *vs* 5m: 0.005916; *p=0.06*; Shannon entropy – baseline: 0.017425 *vs* 5m: 0.011125; *p=0.03*) (Figure 2C).

To analyze the effect of therapy on quasispecies population over time, the mean of genetic distances between each time point since treatment outset and the baseline sample were also calculated (Table 1). Differences among groups of response were compared. Results showed that post-therapy populations of ETR patients (0.0316) presented significantly higher diversity from baseline variants than NR patients (0.0222) (*p=0.006* (Figure 2D). When during and after-therapy variants of NRs were compared to ETRs post-treatment variants, results were also statistically different (0.0205 and 0.0316, respectively; *p=0.000)*.

**Figure 3:**
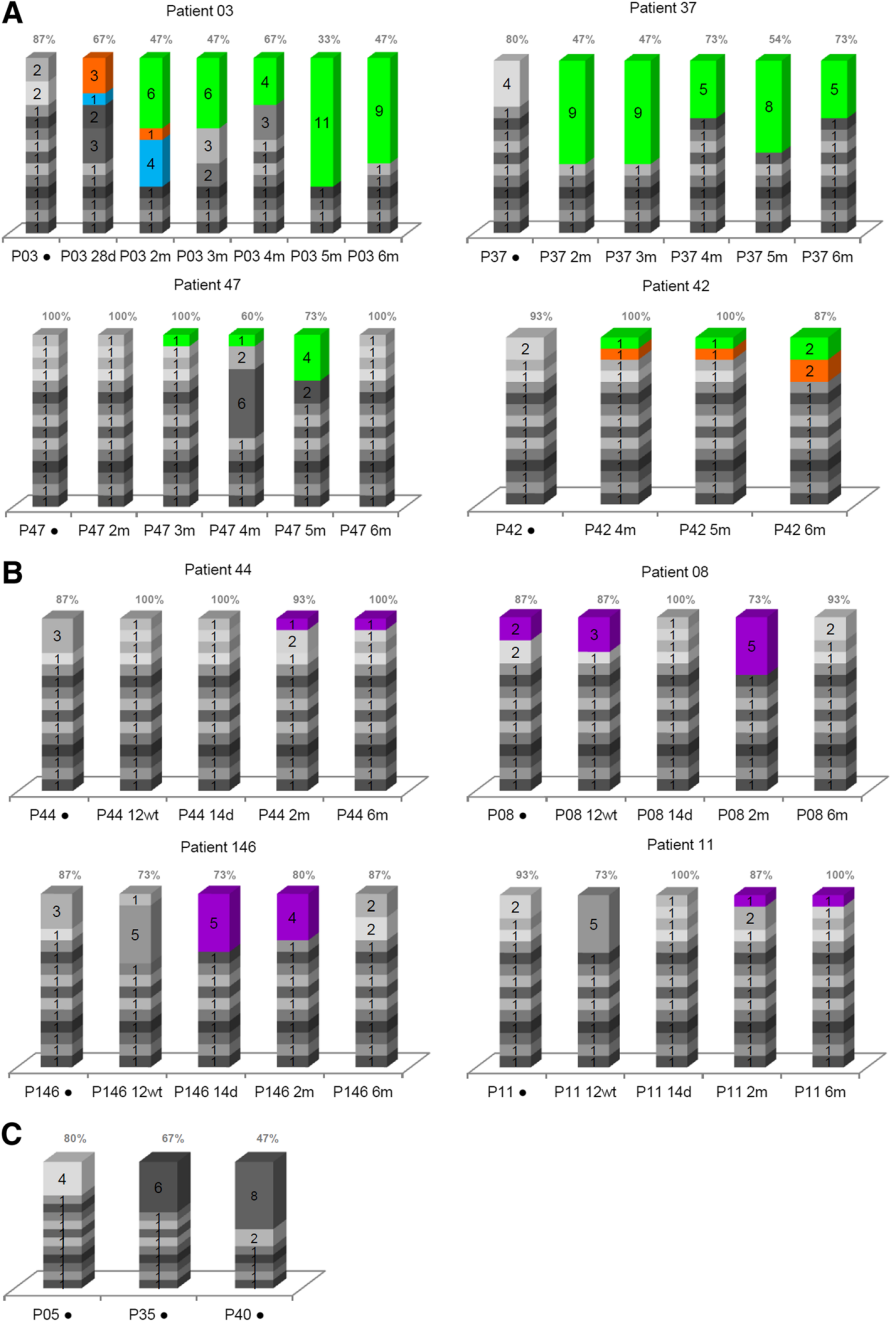
**Quasispecies diversity within the NS5A protein based on the number of strains. A**) The end-of-treatment responders. **B**) The non-responders. **C**) The sustained virological responders. Vertical bars represent viral variants. Inside the bars are the numbers of identical clones and, consequently, the number of identical quasispecies. The predominant quasispecies identified from the samples of each patient are identified by specific colors. The percentages of different quasispecies in ETR, NR and SVR samples are presented above the bars. The time of sample collection are indicated below the bars.

In order to evaluate the number of NS5A quasispecies in each sample, all sequences in this study were analyzed using LOCQSPEC 1.0 software. Figure 3 presents LOCQSPEC 1.0 analyses for ETR and NR patients. The percentages of different quasispecies in ETR, NR and SVR samples are presented.

There was no specific profile of quasispecies diversity. However, samples from ETR patients showed a more varied quasispecies composition before treatment and a more homogeneous composition in several of the after-treatment samples. In some cases, the homogenization of quasispecies composition appeared to be related to the appearance of predominant quasispecies (Figure 3A). The NR samples presented with a more heterogeneous quasispecies composition than the ETR patient samples. The P08 was the only patient in whom the same quasispecies were identified in before- and after-treatment samples (Figure 3B).

The SVR samples contained the most homogeneous quasispecies composition when compared with ETR and NR samples collected before treatment (Figure 3C).

### NS5A quasispecies experience genetic evolution over time

**Figure 4:**
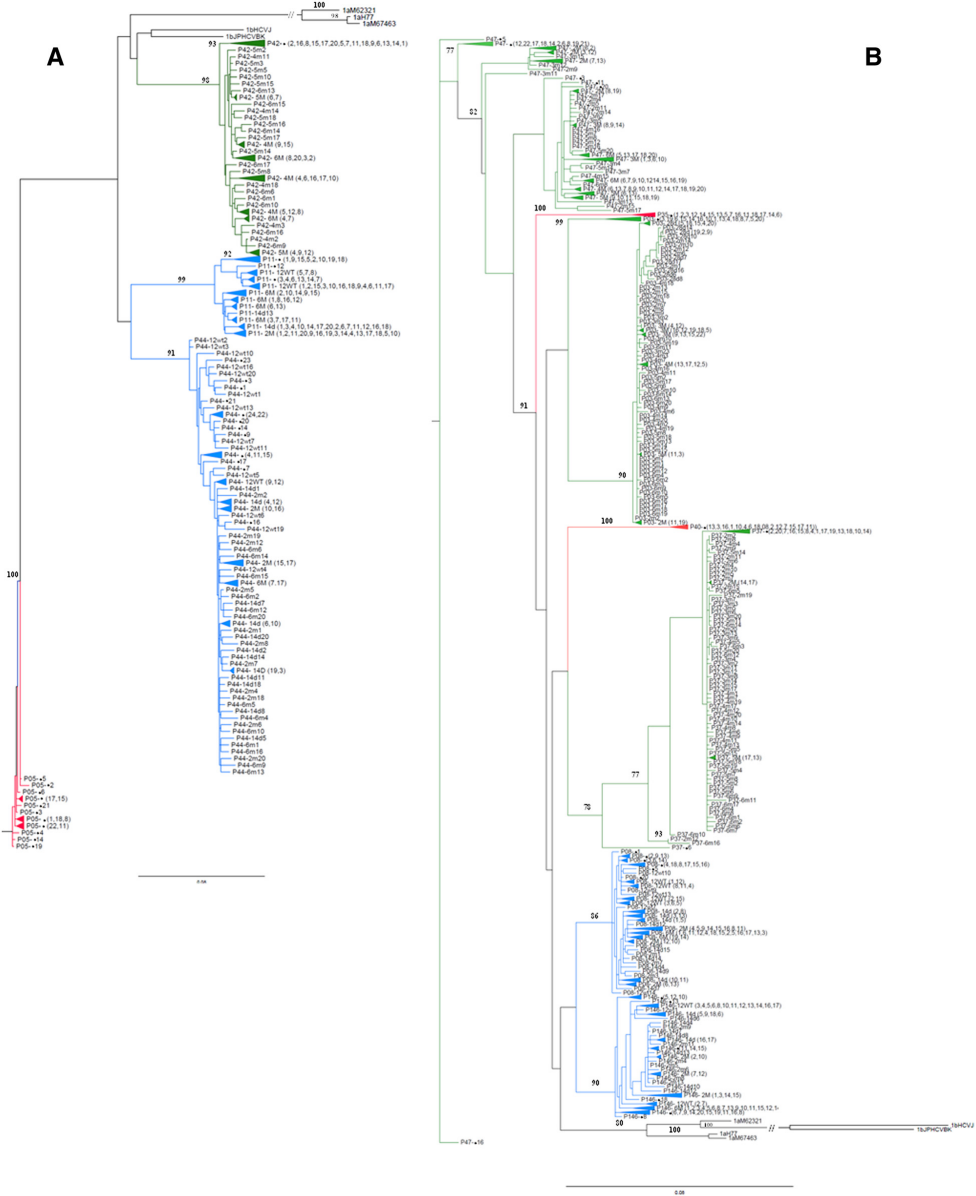
**Unrooted phylogenetic trees reconstructed from the complete NS5A region. **Phylogenetic tree reconstructed using the maximum-likelihood (ML) method, a heuristic search with Nearest Neighbor Interchange (NNI) branch-swapping algorithm. **A**) HCV genotype 1b tree was based on the TVMef+G substitution model. **B**) HCV genotype 1a tree was based on the GTR+G substitution model. References are utilized in both trees. Patterns of response are represented by colors: sustained virological responders in red, non-responders in blue and end-of-treatment responders in green.

Phylogenetic trees were reconstructed from 690 full-length NS5A sequences and reference sequences as described in the Methods section. Sequences clustered according to subtype, presenting monophyletic clusters with genotype 1a or 1b references. Viral isolates corresponding to the same patient samples also clustered, showing they were more closely related than other patient isolates (Figure 4). The topology of sequences from each patient was individually analyzed to evaluate the phylogenetic relationship among isolates. In general, the phylogenetic analysis suggested clustering of isolates of samples collected before treatment from ETR patients. The isolates of samples collected at different times after treatment were mixed and grouped into another cluster. (Additional files 1, 2, 3, 4). Before treatment, quasispecies clustering was observed for P146 from NR patients. Sequences from samples collected before treatment from patients P44, P11 and P08 tended to group together with quasispecies identified after 12 weeks of treatment. However, bootstrap values only sustained clusters for patient P11 (bootstrap value of 69%). (Additional files 5, 6, 7, 8).

In some cases, the phylogenetic analysis suggested that isolates identified in samples collected after treatment demonstrated the longest distances from the main nodes (Additional Information).

**Figure 5:**
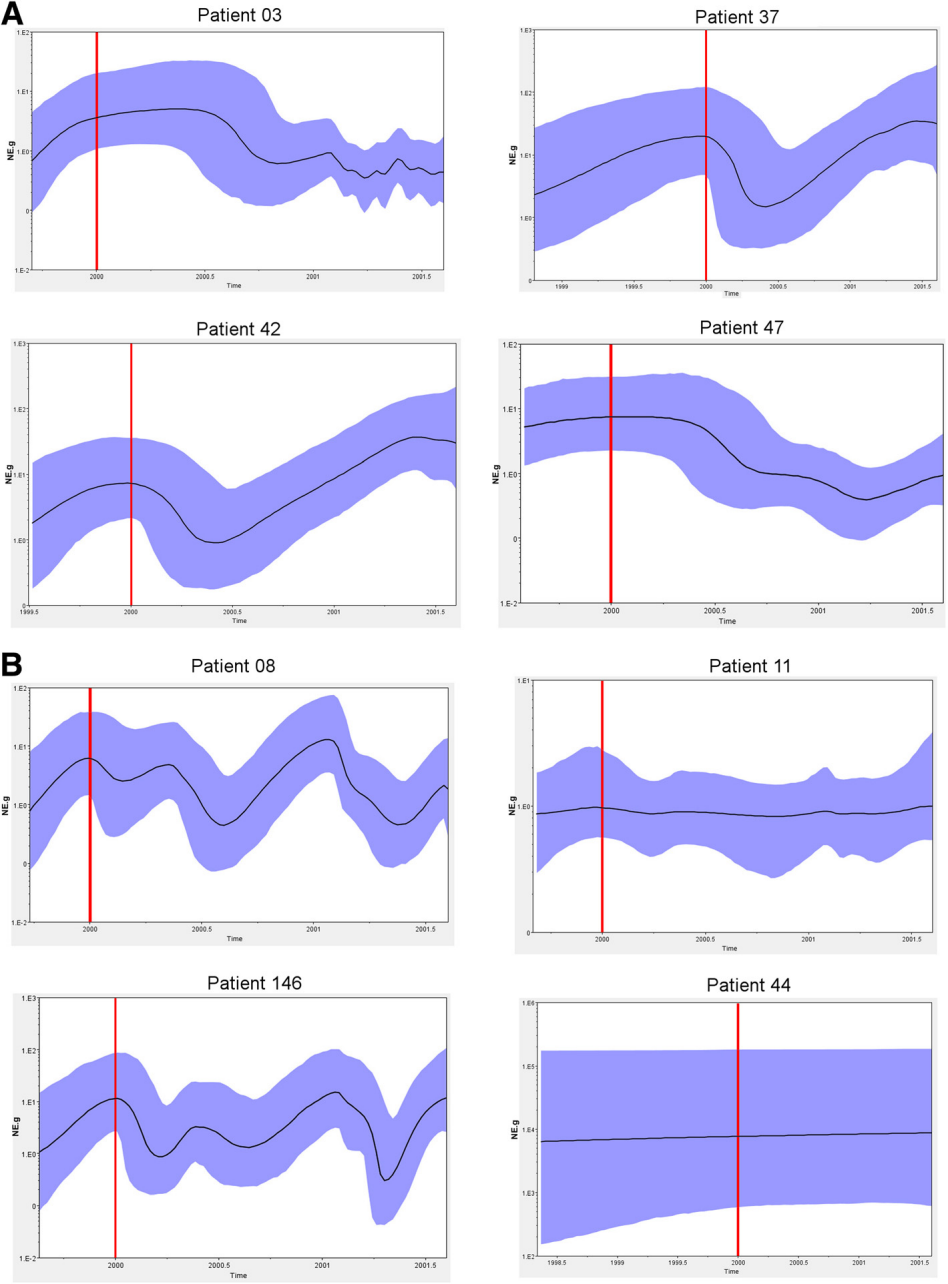
**Effective quasispecies populations among time-point analyzed. **Skyride plots showing the relative genetic diversity of populations from patients ETR (**A**) and NR (**B**). The Black lines represent the median posterior distribution while blue shaded areas are the 95% Bayesian credible intervals. Red lines represent the start of the treatment.

The Skyride analysis shows the variation of effective populations relative to the genetic diversity of each time-point analyzed. In general, a strong decrease of relative populations was observed after therapy for ETR patients. For patients P42 and P37 this variation occurred during the first 6 months showing a trend to recover then. Alternatively, patients P03 and P47 presented a later decrease of post-therapy populations. Non-responders P08 and P146 showed a continuous variation over time that did not seem to be related to therapy and P11 and P44 showed a stability of effective population during all time (Figure 5).

### NS5A quasispecies are under relaxed purifying selective pressure

Phylogenetic trees were reconstructed using DNA alignment of pre and post-therapy NS5A sequences corresponding to each patient in the study. The set of sequences relative to a specific patient was subjected to phylogenetic reconstruction by maximum likelihood. As a result, eight individual phylogenetic trees were generated (Additional files 1, 2, 3, 4, 5, 6, 7, 8). In order to test whether selection pressure varied among different clades, one or two clades of interest were selected for testing. Clades with predominant quasispecies and those in which isolates identified in samples collected after treatment that demonstrated the longest distances from the main nodes were selected, making the assumption that the clade of interest would demonstrate different values of ω (dN/dS) from other branches of the tree. For patient P37, it was not possible to select any specific clade; the detailed results are presented in Table 2. The values of ω ranged among patients from ω = 0.1101 to ω = 0.7177, and the majority of values indicated a purifying selection or relaxed purifying selective pressure. For patients P47 and P11, the foreground ω values were statistically different from the background ones. For these patients, two clades were selected for analysis using phylogenetic topology. For patient P47 these were clade one, where post-therapy quasispecies demonstrated the longest distances, and clade two, where predominant quasispecies were grouped. Accordingly, their ω values were different (clade 1: ωF = 0.2487, 2Δl = 6.4343, and clade 2: ωF = 0.7177, 2Δl = 21.4983). For patient P11, clade one, where post-therapy quasispecies were located, and clade two, where quasispecies from the first sample collected after the end of therapy were grouped, were selected for analysis. The ω values were different for each of these clades (clade 1 ωF = 0.2685, 2Δl = 9.264, and clade 2 ωF = 0.5783, 2Δl = 11.8750). These results indicate a relaxed purifying selection, with the exception of ωF = 0.7177, which approximates to neutral evolution.

**Table 2:** Log likelihood values and parameter estimated under two models of selection pressure within particular clades of NS5A quasispecies

**Patient**	**Model**	**l**	**2Δl**	**Estimates**
**P08**	one-ratio	−4404.76677		**ωB = 0.1692**
	two-ratio	−4404.28734	0.9587	ωB = 0.1521
				ωF = 0.1872
**P11**^a^	one-ratio	−4558.41000		ωB = 0.1835
	two-ratio	−4552.47248	**11.8750	ωB = 0.1650
				**ωF = 0.5783**
	two-ratio	−4553.77795	**9.264	ωB = 0.1419
				**ωF = 0.2685**
**P146**	one-ratio	−4755.95340		**ωB = 0.1705**
	two-ratio	−4755.92063	0.0822	ωB = 0.1727
				ωF = 0.1618
	two-ratio	−4754.96926	1.9683	ωB = 0.1818
				ωF = 0.1246
**P44**	one-ratio	−6356.59235		**ωB = 0.1433**
	two-ratio	−6356.28458	0.6155	ωB = 0.1338
				ωF = 0.1521
**P03**	one-ratio	−3854.07081		**ωB = 0.2003**
	two-ratio	−3853.33452	1.4726	ωB = 0.1977
				ωF = 0.2350
**P42**	one-ratio	−5111.91233		**ωB = 0.1101**
	two-ratio	−5111.91231	0.0004	ωB = 0.1102
				ωF = 0.1101
**P47**^a^	one-ratio	−5455.19420		ωB = 0.2013
	two-ratio	−5451.97704	*6.4343	ωB = 0.1604
				**ωF = 0.2487**
	two-ratio	−5444.44505	**21.4983	ωB = 0.1776
				**ωF = 0.7177**

Non-responder: P08, P11, P146, P44; End-of-treatment responder: P03, P42, P47.

*X*^2 ^critical values, 1 df: *: 3.84; **: 6.63; 2Δ ℓ = 2 (l_1_-l_0_).

The ω values considered are presented in bold.

^a ^Two clades selected for analyses.

Analysis of selection pressure per site also revealed that populations were mainly under purifying selection and no significant difference between response groups was observed regarding the number of sites selected (NRs 106 and ETRs 103; ω < 1). Sites under positive selective pressure were found at codons 157 (P03), 182 and 440 (P42), 62 and 404 (P44). Data not showed.

## Discussion

Quasispecies composition appears to be important in the IFN resistance mechanisms [29], and genetic variability has been studied mainly in E2 and NS5A regions of the HCV genome [30-35]. Additional investigation of NS5A region variability may be relevant as heterogeneity in this region before treatment has been related to IFN-monotherapy responsiveness. However, studies have presented controversial data and the mechanism underlying how NS5A interferes with responsiveness to treatment has yet to be elucidated.

In the present study, the genetic variability of the complete NS5A region in patients infected with HCV genotype 1 was analyzed. Altogether, 690 sequences of the entire NS5A from 11 patients’ samples were analyzed. Samples showed lower genetic diversity of quasispecies composition at baseline for SVR patients than NR and patients who relapsed after the end of therapy (ETR). Those significant differences among genetic distance of before treatment were not observed in our previous work when we exclusively analyzed baseline samples [36]. However, the group of patients and sequences analyzed were not identical, being only part of sequences of previous work used to these analyses.

Our findings are consistent with previous observations made on patients undergoing anti-HCV therapy that less complexity and lower diversity of HCV quasispecies at the baseline is associated with viral clearance [19,21,37-39]. Therefore, high genetic variability of HCV could be related to the low efficiency of anti-HCV treatment [22]. Jain et al. observed that the initial anti-viral effect of interferon is influenced by the quasispecies composition at the time of treatment initiation, and patients who demonstrated high viral diversity were less likely to respond to treatment [21]. In agreement with the genetic distance and entropy results, the number of viral strains at baseline was lower for SVR patients than NR and ETR patients, showing that quasispecies composition was more homogeneous than other groups of responses. A recent study evaluating the clinical and virological parameters that could be associated with or predictive of therapy outcome at baseline demonstrated that a higher number of quasispecies variants in the E1/E2 region was significantly associated with treatment failure. However, it is inferred that independent factors cannot provide a consistent prediction of therapy response [40].

In order to investigate whether therapy caused a significant change in the population over time, we compared variants heterogeneity of baseline, during therapy and follow-up by analyzing the mean of genetic distance and Shannon entropy among each time-point. No specific profile of quasispecies diversity for all patients from each response type could be identified over time. However, significant differences were observed when baseline and post-therapy variants of NRs and ETRs were compared. In either case post-therapy population were significant less heterogeneous than baseline showing a decrease of diversity over time.

We also calculated the mean of genetic distances between each two time points and could observe that when compared to the baseline, post-therapy populations of ETR patients presented significantly higher diversity from baseline variants than NR patients.

Those findings correlate with data obtained by tracking the number of strains. Analyzing ETR, it was evident that quasispecies composition was more homogeneous in post-therapy samples. This homogeneity was most evident in patients P37 and P03, who presented with homogeneous quasispecies composition from the rebound and sustained it. As with ETR patients, the HCV RNA is undetectable by the end of therapy, suggesting that the selective pressure of treatment acts on quasispecies diversity during it until HCV becomes undetectable in samples. One explanation is that during or after the end of therapy predominant quasispecies rise in frequency, while others decrease progressively in frequency or are eliminated. The persistence of this predominant variant could be favorable for sustaining the infection, causing it to become chronic again.

Farci at al. evaluated the quasispecies composition of the E1/E2 region in samples from patients who were untreated or treated with conventional IFN combined with ribavirin. Analyzing samples of relapse patients, they observed greater viral diversity at baseline, and an emergence at relapse of a new dominant strain, or the emergence of a minor dominant population [41]. They inferred that the emergence of a new viral strain after rebound implies that the majority of baseline variants were sensitive to IFN and that the origin of these new strains is uncertain. The authors concluded that it is probable that very low levels of virus replication continued to occur despite the disappearance of viremia [41].

A study focusing on breakthrough response pattern, defined by patients who have an initial response followed by reactivation whilst receiving IFN therapy, did not identify quasispecies variants from breakthrough in baseline samples [42]. This study assumed that HCV variants sampled at the time of breakthrough represent drug-resistant quasispecies, and if present at baseline, they must be present very low frequency. According to the authors viral breakthrough could be attributed to the selection of pre-existing drug-resistant variants or the emergence of different quasispecies with reduced sensitivity to IFN. However, the study indicated that selection is mostly responsible for appearance of drug resistant quasispecies at breakthrough [42]. As breakthrough and relapse indicate rebound of infection after undetectable HCV RNA levels, is reasonable that the results presented herein are in agreement when considering the quasispecies composition and diversity at breakthrough and relapse time.

The data collected from non-responder samples revealed a very diverse composition of viral strains over the time course of the investigation. As described previously, the high variability of the quasispecies population in these patient samples might represent a continuous process of adaptation [43], as these variants were continuously eliminated during and after the end of treatment in most cases. Alternatively, it could offer the virus some advantage in sustaining the infection as a large number of quasispecies may indicate a better opportunity for virus persistence [44,45].

The analysis of the number of effective populations among time-point analyzed showed different profiles for the types of response. A strong decrease of effective populations was observed after therapy for ETR patients whereas NRs presented a continuous variation or stability of its populations over time. Those data are in agreement to nucleotide diversity results where ETRs demonstrated a decrease of population number and heterogeneity presenting more distinct post-therapy variants. In general, populations of those patients experience a bottleneck phenomenon after therapy. On the other hand, profiles found to NRs assent to the continuous high diverse population and a lower diversity between pre and post-therapy variants probably due to a minor effect of treatment in variants selection. Pawlotsky et al. compared pre- and post-therapy NS5A amino acid sequences from non-responder patients and demonstrated that most variants from after-treatment samples were not detected before treatment, whereas most pre-treatment variants were no longer evident after treatment [22]. The data presented here agree with their findings, with the exception of samples from patient P08. In the P08 samples, a variant was identified at baseline (13% of NS5A amino acid sequences) that was detected after 12 weeks of treatment and two months after therapy was completed. Several studies have showed that variants resistant to therapy may be present at baseline, and it is proposed that the persistence of these variants in patients who fail to respond suggests the existence of virus strains with inherent resistance to IFN [41,43].

Consistent with previous observations, phylogenetic analysis of virus sequences obtained from patients failed to show any clustering associated with specific response [34,41,46]. However, the data suggested that pre- and post-therapy isolates from samples of ETR patients tended to group together in different branches. For NR samples, before-treatment quasispecies variants tended to group with quasispecies found after 12 weeks of treatment, and post-therapy sequences grouped together in another branch. The distinctive clustering of pre/post-therapy sequences had been described previously [22,42,47], demonstrating the evolutionary process occurred over time. In some cases, isolates identified in after-treatment samples had the longest distances from the main nodes in phylogenetic analysis. This may suggest that changes can improve the fitness of quasispecies resulting in a persistent infection after treatment selection pressure.

To investigate whether quasispecies were under differential selective pressure over time, we performed analysis with the set of sequences of each NR and ETR patient. In general, the results showed that a purifying selection is driving the evolution of quasispecies over time. In some cases, the purifying selection was relaxed (clades selected for patients P11 and P47). In such cases, two scenarios could be envisaged. Variants undetected in the baseline sample due its low frequency could increase in frequency under relaxed selective pressure and consequently be detected in the after therapy samples for some time. Alternatively, those variants could have arisen by mutation during treatment increasing in frequency and being detected then. Relaxed purifying selection may also be indicative of some codons of the entire protein being under positive selection. However, we failed to identify sites under positive selection for those patients. Codons under positive selective pressure observed in this work could not be related to the therapy outcome and were not present in preferential position in the NS5A protein. A study analyzing PKR binding domain and the V3 domain in the NS5A region identified positively selected sites but also failed to detect a pattern mechanism for the inefficient response to anti-viral treatment [48]. Therefore, further analyses are necessary.

Genetic analyses identified nonsense mutations in the NS5A sequence of some HCV variants. Nonsense mutations in samples from HCV-infected patients have been described [34,36,49], but the structural and functional implications of such mutations detected in vivo are unclear. Most of the nonsense mutations detected were located in the N-terminal region of NS5A (Alpha Helix, Domain I or Low Complexity Sequence I regions of the NS5A); only one was located in the C-terminal region (domain III). It is known that N-terminal mutants of NS5A are preferentially located in the nucleus and are reported to function as transcriptional regulators. A recent study using a HCV replicating cell system revealed that during the life cycle a variety of N-terminally truncated NS5A fragments are generated [50]. Tests on several of these truncated NS5A fragments demonstrated that they were preferentially located in the nucleus or equally distributed between the cytoplasm and nucleus. The full length NS5A (1–449) was located in the extra-nuclear compartment as previously described. However, truncated forms impaired HCV replication. In contrast, domain III of the C-terminal NS5A region can be deleted with no or minimal effect on RNA replication [51,52], but the C-terminal region residing between amino acid residues 2404 and 2435 of domain III is crucial for virus production [53]. To elucidate the effects of nonsense mutations identified in the present study, further analysis with HCV replicating cells is necessary. Truncated NS5A forms observed in previous studies show drawbacks in terms of replication or assembly, but these effects could be overcome by the diversity of quasispecies composition.

## Conclusions

This study confirms that heterogeneous diversity of quasispecies pre-therapy could be related to a low response to IFN-based therapy, and that homogeneity of quasispecies composition at baseline with viral clearance.

The follow-up of patients’ samples showed that genetic diversity of populations decreased over time. Post-therapy population of end-of-treatment responders presented higher genetic distance from baseline probably due to the bottleneck phenomenon observed for those patients in the end of treatment. The effective population of those patients also showed a strong decrease after therapy. Otherwise, NRs demonstrated a continuous variation or stability of effective populations and genetic diversity over time that did not seem to be related to therapy. Quasispecies distribution of NS5A was variable and distinctly clustered over time. In addition, the evolution of quasispecies over time was subjected to purifying or relaxed purifying selection showing that the majority of the synonymous mutations are not being fixed. Some codons were found to be under positive selective pressure but it failed to be related to the therapy.

Therefore, the quasispecies composition and evolution over time are factors to be considered in terms of patient outcome after combined therapy for chronic hepatitis C.

## Methods

### Patients

Eleven naïve patients infected chronically with HCV RNA genotypes 1a or 1b were enrolled from the Hepatology Department of the São José do Rio Preto School of Medicine. Patients with other concomitant liver diseases [hepatitis B virus (HBV) or other hepatotropic virus infections, alcohol abuse, autoimmune hepatitis and hereditary liver diseases] were excluded. This study was approved by “The Ethics Committee of the School of Medicine of São José do Rio Preto”, and written informed consent was obtained from all patients who consented to their individual data being included in the manuscript. Plasma samples were collected before, during and after the end of treatment. During the 48-week treatment, patients received PEG-IFN-α-2b (according to body weight) subcutaneously once a week and RBV daily, taken orally at a dose of 600–1,200 mg (according to body weight).

The patients were classified into three groups according to their response to therapy: three patients demonstrated sustained virological response (SVR); four patients were non-responders (NR) and four patients were end-of-treatment virological responders (ETR) (Table 1). SVR was defined as absence of HCV RNA in plasma using qualitative PCR six months after the end of therapy. NR was defined as continued presence of HCV RNA in plasma during treatment and six months after the end of treatment. Patients who were HCV RNA-negative at the end of therapy but experienced a relapse were classified as ETR. Breakthrough response pattern defined by patients who have an initial response followed by reactivation whilst receiving IFN therapy was not analyzed in this study based on time point analyzed during treatment.

For all patients, one sample before treatment was analyzed. Additional samples were collected during therapy (12 weeks of therapy) and after the end of therapy (14 days, two and six months) for non-responders, and at the relapse time and then monthly for end-of-treatment responders. Samples analyzed for each patient are detailed in Table 1. Samples from the SVR group and before treatment samples from most patients (P05, P35, P40, P44, P03, P37 and P42) enrolled in this study were analyzed in a previous work (identification of patients: P2, P3, P4, P8, P9, P10 and P11 respectively) and were used in this study for comparative analyzes of the evolutionary dynamic of quasispecies [36]. Baseline sample of patients P08, P11, P146 and P47 analyzed in this work were not previously done. Additionally, all samples collected during and after the end of treatment were exclusively analyzed in this work.

### RNA extraction, RT-PCR and NS5A amplification

Total RNA was extracted from 140 μl of plasma using the commercially available QIAamp Viral RNA Kit (Qiagen, Uniscience). RNA was reverse-transcribed into cDNA using a High-Capacity cDNA Archive kit (Applied Biosystems, Foster City, CA, USA) and random primers. The mixture was incubated at 37°C for two hours. For amplification of the entire NS5A region of the HCV genome, a nested polymerase chain reaction (PCR) was performed using the primers described previously [36].

The viral load was quantified using the Cobas TaqMan HCV Test according to the manufacturer’s instructions.

### Cloning and sequencing

PCR products of approximately 1.7 kb were purified and ligated into the pCR-XL-TOPO-vector using the TOPO XL PCR cloning kit (InvitrogenTM Life Technologies, Carlsbad, CA, USA). The ligation products were transformed into competent cells (InvitrogenTM Life Technologies, Carlsbad, CA, USA). Fifteen transformants were randomly chosen for further studies, and plasmid DNA was isolated from a 3.0-ml broth culture using the GeneJET plasmid Miniprep Kit (Fermentas). Recombinant pCR-XL-TOPO-NS5A clones were sequenced using dideoxy terminator automated sequencing (ABI Prism Ready Reaction Mix; Applied Biosystems, Foster City, CA, USA) using an ABI Prism 377 and ABI 3130XL sequencers, according to the manufacturer’s instructions (Applied Biosystems Inc, Foster City, CA, USA.). Eight to ten sequencing reactions were performed for each clone, using flanking primers, M13 Forward and M13 Reverse (Invitrogen TM Life Technologies, Carlsbad, CA, USA), internal forward primers (1aF1 5’CACCAGTGGATAASCTCGGA3’, 1aF2 5’CCCATYAATGCCTACACCAC3’, 1aF3 5’CTGTCYGCTCCATCTCTCA3’ and 1aF4 5’GARTCAGARAACAAAGTGGTG3’ for genotype 1a; 1bF1 5’ATCCTCTCHAR-CCTTACCAT3’, 1bF2 5’ GRACATTCCCCRTCAACGC3’, 1bF3 5’ 5’GTCCTRACAG-AATCCACMGTG3’ and 1bF4 5’CCARTTGTCTGCGCCTTC3’ for genotype 1b) and internal reverse primers (1aR1 5’ACATWGAGCAACACACGAC3’, 1aR2 5’GTTCCC-TTGAGAGATGGAGC3’ and 1aR3 5’TAGGCATTRATGGGGAAGGT3’ for genotype 1a; 1bR1 5’ARCARCAGACGACGTCCTC3’, 1bR2 5’AGCGGGTCGAAAGAGTCCA 3’ and 1bR3 5’ GAACCGTTTTTGACATGTCC3’ for genotype 1b).

### Genetic and Evolutionary Analysis

All sequences were analyzed with Phred-Phrap programs [54-56]. These programs analyze the quality of the sequences and align them in complete NS5A contigs. This study generated 585 sequences of full length NS5A from samples collected during and after treatment and for patients P08, P11, P146 and P47 also from the sample collected before the beginning of the treatment. In order to analyze viral evolution along time, 105 sequences from samples collected before the treatment, previously published (Accession numbers: EU309511 - EU309525, EU309586 - EU309599 and EU309600 - EU309614) [35], were used in the analyses. Consequently, 690 full-length NS5A sequences were analyzed in this study, corresponding to 11 patients. The nucleotide sequence contigs (1344 nucleotides for genotype 1a and 1341 for genotype 1b) were aligned using the Clustal X program (version 1.81) [57] and amino acid sequences were obtained. Primer sequences were removed from all sequences using the BioEdit program (version 7.0.5.3) [58]. The genetic distance between pairs of sequences were calculated with MEGA version 4 or 5 using the p-distance or Tamura Nei methods [59]. The variability of amino acid residues in each position (i) was measured by calculating the Shannon entropy [22]. The entropy value is a measure of the lack of information at each position of the amino acid sequence. The Shannon Entropy was calculated at the amino acid level as follows: H(i) = −(Σf(b,i)log(base 2)f(b,i)), where f(b,i) is the frequency with which each residue b appears in position i of the protein. When different groups of virological response were compared, the value was normalized by Sn=H/logN, where N is the total number of sequences analyzed in each sample [58].

For analyzing quasispecies variability, all sequences of complete NS5A generated in this study were analyzed using software LOCQSPEC 1.0 [60] and the contigs that presented the same nucleotide or amino acid sequences were grouped together. Bayesian skyride plots were performed using the BEAST package [61].To construct the phylogenetic tree of NS5A variants obtained from the patient samples, the PAUP* version 4 program was used [62]. Phylogenetic trees were constructed using the maximum likelihood method with the model of substitution, as determined by hierarchical likelihood ratio test score criteria in Modeltest 3.06 [63]. Base frequency, gamma distribution and transition/transversion ratios were determined (from the data) by Modeltest 3.06. A thousand replicates were used to test the support given by the data to the clusters of the tree topology, and bootstrap values >70 were considered significant [64]. Genetic and phylogenetic analyses were performed using the standard genotype 1a sequence H77 (NC_004102.1) as a reference and genotype 1b sequence HCV-J (D90208.1), obtained from GenBank. The ratio between the relative rate of non-synonymous substitution to the relative rate of synonymous substitution (ω=dN/dS) measures the strength of selection acting on a protein-coding gene. Assuming synonymous mutations are subjected to almost strictly neutral selection, ω<1, ω=1, and ω>1 represent negative selection, neutral evolution, and positive Darwinian selection, respectively [65]. Site per site ω was calculated by Single Likelihood Ancestor Counting (SLAC) method using HyPhy [66]. Maximum likelihood analysis of the sequence evolution was performed using the CODEML program in the PAML 3.15 software package [67]. Initially, phylogenetic trees were reconstructed using the maximum likelihood method and the HKY model of substitution, as determined by hierarchical likelihood ratio test score criteria in Modeltest 3.06 for each dataset of NS5A sequences corresponding to each non-responder and end-of-treatment responder in this study. The ratios of global synonymous changes per site (dS) versus replacement changes per site (dN) for each tree were calculated using two models described by Yang [68]. The one-ratio model assumed an equal ω ratio for all branches in the phylogeny. The two-ratio model assumed two ω ratios: one branch for the background (ω B), one for the foreground branch (ω F = branch of interest) leading to a specific clade of the phylogenetic trees, specified in the Additional Information. This analysis makes different assumptions about the dN/dS ratios for branches of interest relative to the background dN/dS ratio for all other branches. For instance, the “two-ratio” model assumes that the branches of interest have a dN/dS ratio that is different from the background ratio.

### Statistical analysis

The results are presented as mean ± SD, or as percentages. Comparisons among the sustained virological responders, end of treatment responders and non-responders were determined using Tukey’s or Fisher’s statistical test after performing an one-way ANOVA or Chi square. A paired *t*-test was performed to compare means between two time-points. In all tests, a P value less than 0.05 was considered significant.

### Accession numbers

All sequences obtained in this study were submitted to the GenBank nucleotide sequence database (http://www.ncbi.nlm.nih.gov/genbank/). Accession numbers: GenBank:HQ823765 - HQ824349 (sequences obtained in this study), GenBank:EU309511 - EU309525, GenBank:EU309586 - EU309599 and GenBank:EU309600 - EU309614 (sequences generated in a previous study) [35].

## Competing interests

The authors have declared that no competing interests exist.

## Authors’ contributions

ACGJ participated in the design of the study, performed experiments, analyzed the data and drafted the manuscript. RPAM performed experiments. CB, RAS and CMAC analyzed the data and critically revised the manuscript. LHTY analyzed the data. JRRP designed the study and contributed to acquisition and analysis of data. RMF contributed to acquisition and analysis of data. IMVGCM designed the study, analyzed the data and critically revised the manuscript. PR designed and coordinated the study and helped to draft the manuscript. All authors read and approved the final manuscript.

## Pre-publication history

The pre-publication history for this paper can be accessed here:

http://www.biomedcentral.com/1471-2334/13/61/prepub

## Supplementary Material

Additional file 1 **Phylogenetic trees reconstructed from sequences obtained from patient P42 samples.** Maximum likelihood tree reconstructed from full length NS5A region sequences obtained from samples from patient P42 (ETR) plus reference sequence of genotype 1b HCV-J. The number of 1000 permuted trees supporting a clade indicated when that proportion was greater than 70%. The same quasispecies are colored in red or pink. A sequence with nonsense mutation is colored in blue. The clade selected for selective pressure analysis is indicated by a gray line. Click here for file

Additional file 2 **Phylogenetic trees reconstructed from sequences obtained from patient P03 samples.** Maximum likelihood tree reconstructed from full length NS5A region sequences obtained from samples of P03 (ETR) plus reference sequence of genotype 1b HCV-J. The number of 1000 permuted trees supporting a clade indicated when that proportion was greater than 70%. The same quasispecies are colored in red. Sequence with nonsense mutation is colored in blue. The clade selected for selective pressure analysis is indicated by a gray line. Click here for file

Additional file 3 **Phylogenetic trees reconstructed from sequences obtained from patient P47 samples.** Maximum likelihood tree reconstructed from full length NS5A region sequences obtained from samples of P47 (ETR) plus reference sequence of genotype 1b HCV-J. The number of 1000 permuted trees supporting a clade indicated when that proportion was greater than 70%. The same quasispecies are colored in red. Sequence with nonsense mutation is colored in blue. The clades selected for selective pressure analysis are indicated by a gray line (clade 1, ω = 0.2487 and clade 2, ω = 0.7177). Click here for file

Additional file 4 **Phylogenetic trees reconstructed from sequences obtained from patient P37 samples.** Maximum likelihood tree reconstructed from full length NS5A region sequences obtained from samples of P37 (ETR) plus reference sequence of genotype 1b HCV-J. The number of 1000 permuted trees supporting a clade indicated when that proportion was greater than 70%. The same quasispecies are colored in red. Sequence with nonsense mutation is colored in blue. Click here for file

Additional file 5 **Phylogenetic trees reconstructed from sequences obtained from patient P11 samples.** Maximum likelihood tree reconstructed from full length NS5A region sequences obtained from samples of P11 (NR) plus reference sequence of genotype 1b HCV-J. The number of 1000 permuted trees supporting a clade indicated when that proportion was greater than 70%. The same quasispecies are colored in red. The clades selected for selective pressure analysis are indicated by a gray line (clade 1, ω = 0.5783 and clade 2, ω = 0.2685). Click here for file

Additional file 6 **Phylogenetic trees reconstructed from sequences obtained from patient P44 samples.** Maximum likelihood tree reconstructed from full length NS5A region sequences obtained from samples of P44 (NR) plus reference sequence of genotype 1b HCV-J. The number of 1000 permuted trees supporting a clade indicated when that proportion was greater than 70%. The same quasispecies are colored in red. Sequence with nonsense mutation is colored in blue. The clade selected for selective pressure analysis is indicated by a gray line. Click here for file

Additional file 7 **Phylogenetic trees reconstructed from sequences obtained from patient P146 samples.** Maximum likelihood tree reconstructed from full length NS5A region sequences obtained from samples of P146 (NR) plus reference sequence of genotype 1b HCV-J. The number of 1000 permuted trees supporting a clade indicated when that proportion was greater than 70%. The same quasispecies are colored in red. Sequence with nonsense mutation is colored in blue. The clade selected for selective pressure analysis is indicated by a gray line. Click here for file

Additional file 8 **Phylogenetic trees reconstructed from sequences obtained from patient P08 samples.** Maximum likelihood tree reconstructed from full length NS5A region sequences obtained from samples of P08 (NR) plus reference sequence of genotype 1b HCV-J. The number of 1000 permuted trees supporting a clade indicated when that proportion was greater than 70%. The same quasispecies are colored in red. The clade selected for selective pressure analysis is indicated by a gray line. Click here for file
