# Supplementary figures and images for: Analysis of HCV quasispecies dynamic under selective pressure of combined therapy
Source: BMC Infect Dis. 2013 Feb 1;13:61. doi: 10.1186/1471-2334-13-61 (PMC3598780; doi:10.1186/1471-2334-13-61)

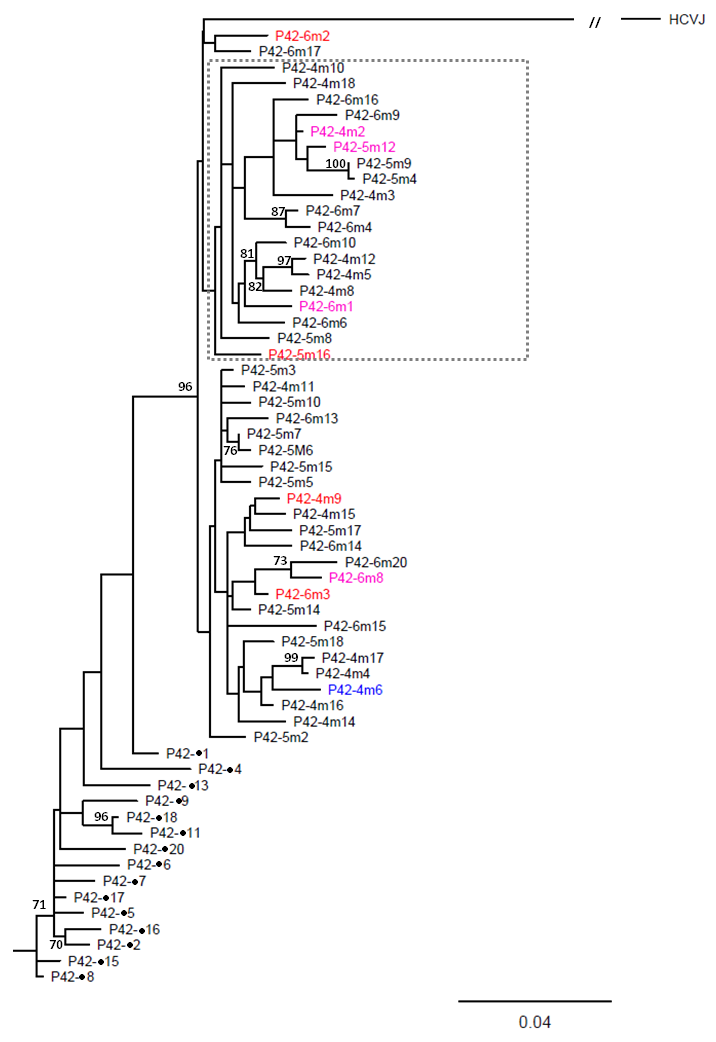

Supplement: Additional file 1 — Phylogenetic trees reconstructed from sequences obtained from patient P42 samples. Maximum likelihood tree reconstructed from full length NS5A region sequences obtained from samples from patient P42 (ETR) plus reference sequence of genotype 1b HCV-J. The number of 1000 permuted trees supporting a clade indicated when that proportion was greater than 70%. The same quasispecies are colored in red or pink. A sequence with nonsense mutation is colored in blue. The clade selected for selective pressure analysis is indicated by a gray line. [file 1471-2334-13-61-S1.tiff]

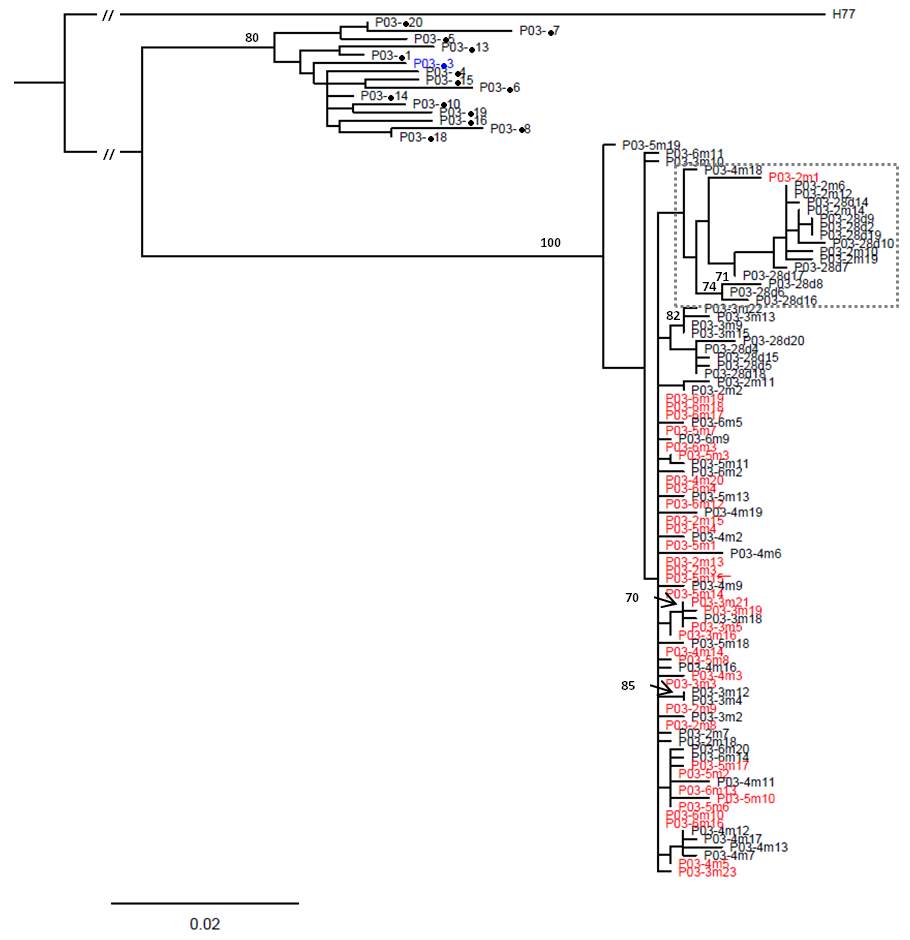

Supplement: Additional file 2 — Phylogenetic trees reconstructed from sequences obtained from patient P03 samples. Maximum likelihood tree reconstructed from full length NS5A region sequences obtained from samples of P03 (ETR) plus reference sequence of genotype 1b HCV-J. The number of 1000 permuted trees supporting a clade indicated when that proportion was greater than 70%. The same quasispecies are colored in red. Sequence with nonsense mutation is colored in blue. The clade selected for selective pressure analysis is indicated by a gray line. [file 1471-2334-13-61-S2.tiff]

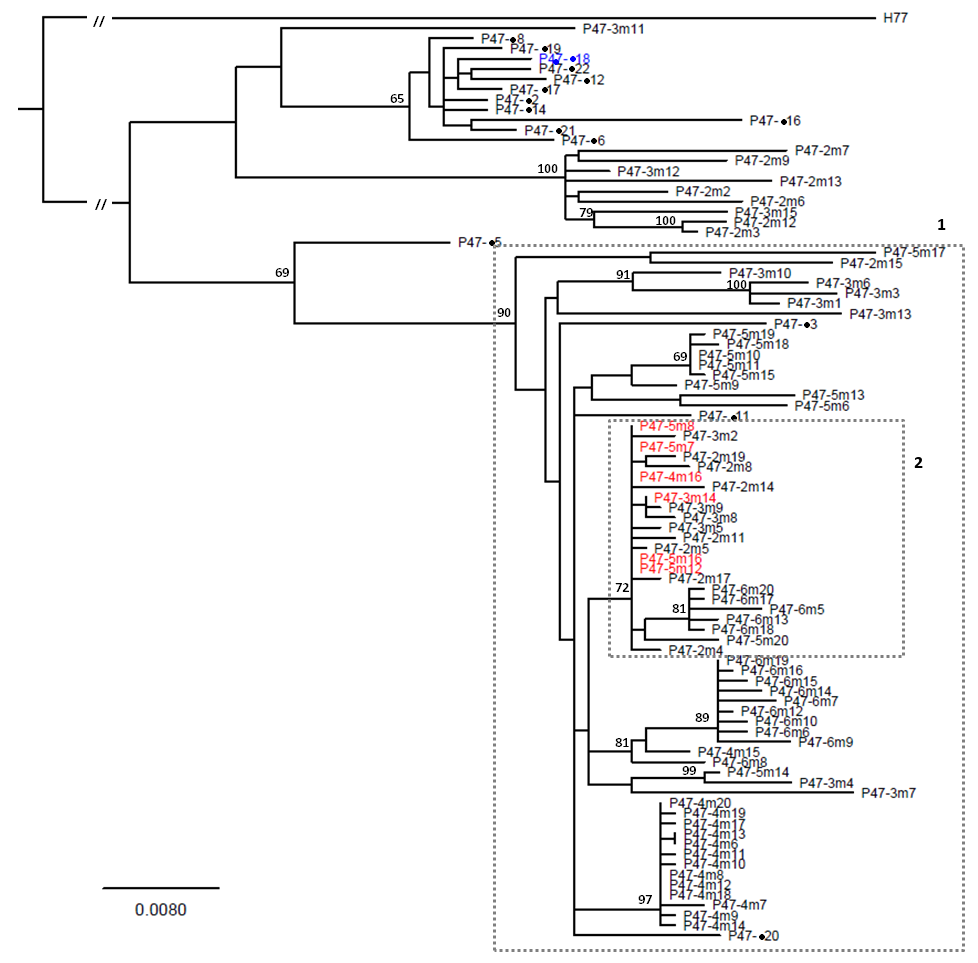

Supplement: Additional file 3 — Phylogenetic trees reconstructed from sequences obtained from patient P47 samples. Maximum likelihood tree reconstructed from full length NS5A region sequences obtained from samples of P47 (ETR) plus reference sequence of genotype 1b HCV-J. The number of 1000 permuted trees supporting a clade indicated when that proportion was greater than 70%. The same quasispecies are colored in red. Sequence with nonsense mutation is colored in blue. The clades selected for selective pressure analysis are indicated by a gray line (clade 1, ω = 0.2487 and clade 2, ω = 0.7177). [file 1471-2334-13-61-S3.tiff]

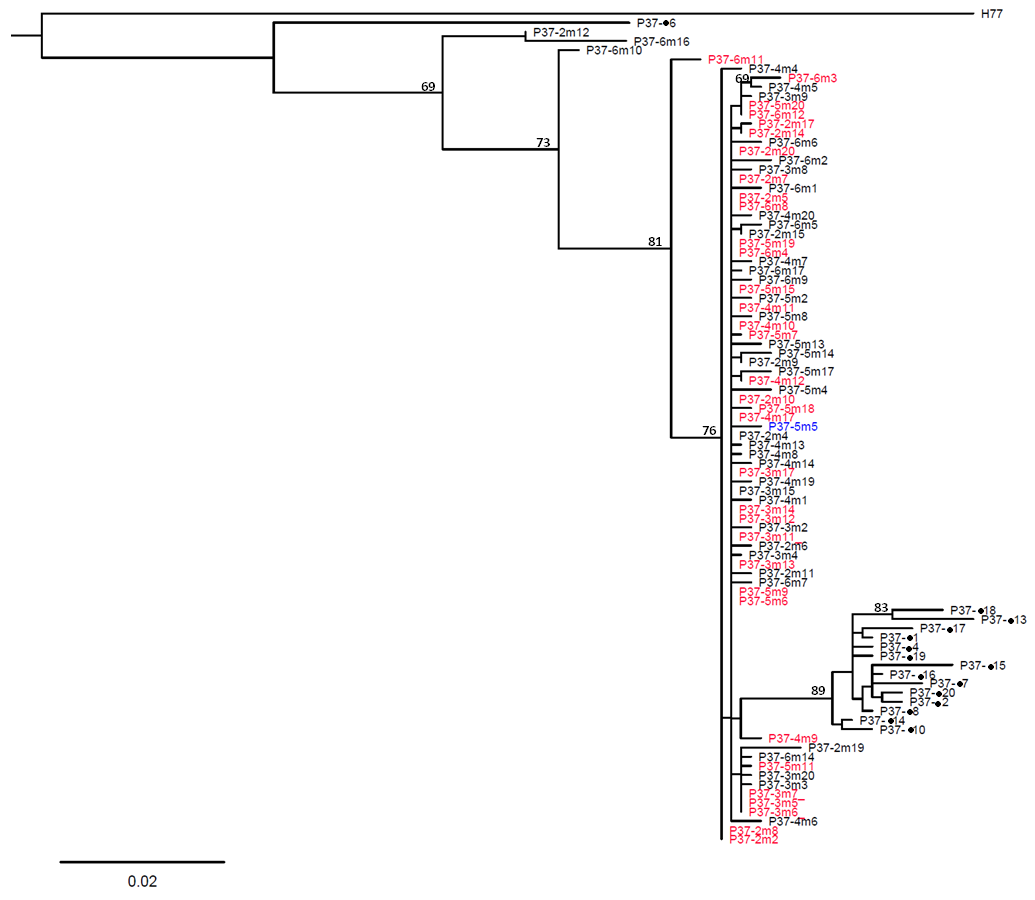

Supplement: Additional file 4 — Phylogenetic trees reconstructed from sequences obtained from patient P37 samples. Maximum likelihood tree reconstructed from full length NS5A region sequences obtained from samples of P37 (ETR) plus reference sequence of genotype 1b HCV-J. The number of 1000 permuted trees supporting a clade indicated when that proportion was greater than 70%. The same quasispecies are colored in red. Sequence with nonsense mutation is colored in blue. [file 1471-2334-13-61-S4.tiff]

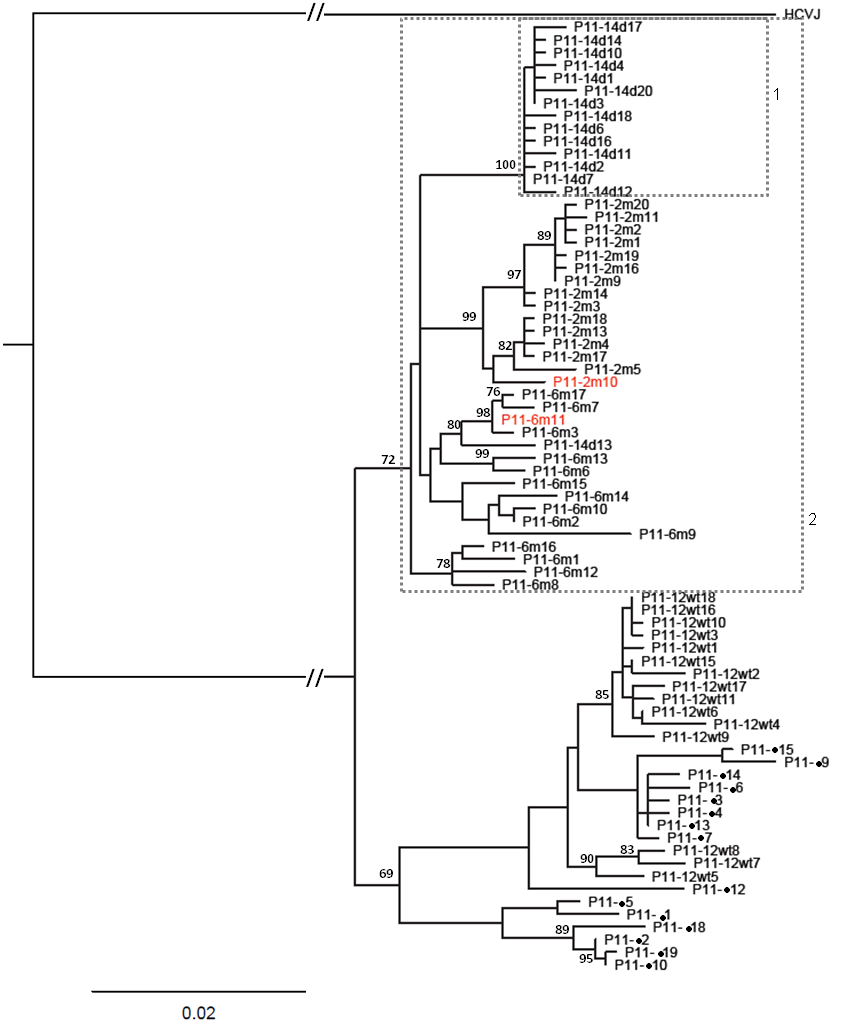

Supplement: Additional file 5 — Phylogenetic trees reconstructed from sequences obtained from patient P11 samples. Maximum likelihood tree reconstructed from full length NS5A region sequences obtained from samples of P11 (NR) plus reference sequence of genotype 1b HCV-J. The number of 1000 permuted trees supporting a clade indicated when that proportion was greater than 70%. The same quasispecies are colored in red. The clades selected for selective pressure analysis are indicated by a gray line (clade 1, ω = 0.5783 and clade 2, ω = 0.2685). [file 1471-2334-13-61-S5.tiff]

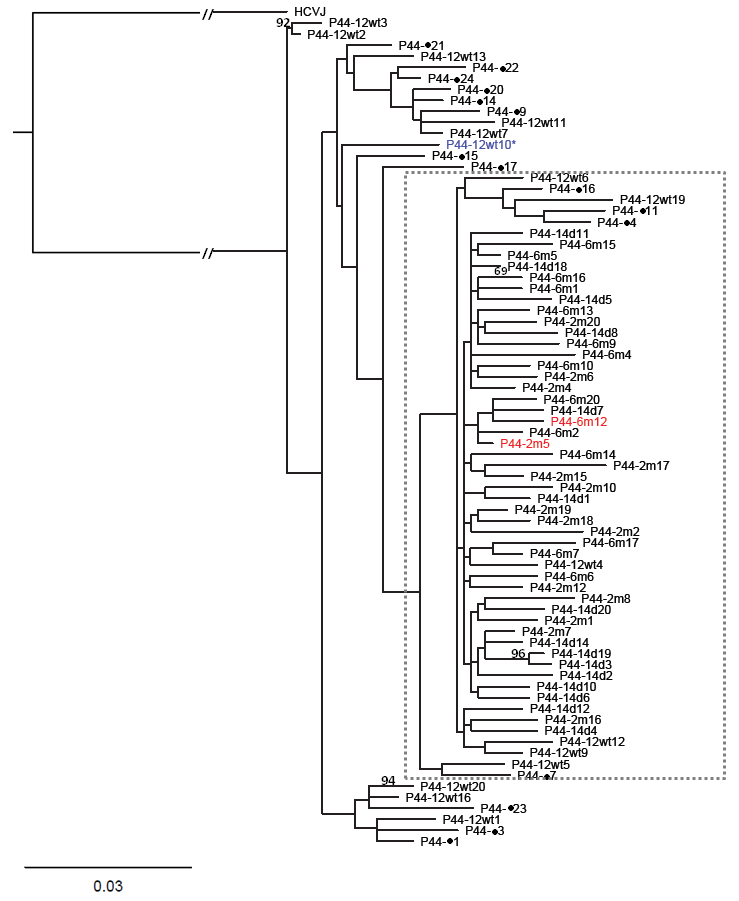

Supplement: Additional file 6 — Phylogenetic trees reconstructed from sequences obtained from patient P44 samples. Maximum likelihood tree reconstructed from full length NS5A region sequences obtained from samples of P44 (NR) plus reference sequence of genotype 1b HCV-J. The number of 1000 permuted trees supporting a clade indicated when that proportion was greater than 70%. The same quasispecies are colored in red. Sequence with nonsense mutation is colored in blue. The clade selected for selective pressure analysis is indicated by a gray line. [file 1471-2334-13-61-S6.tiff]

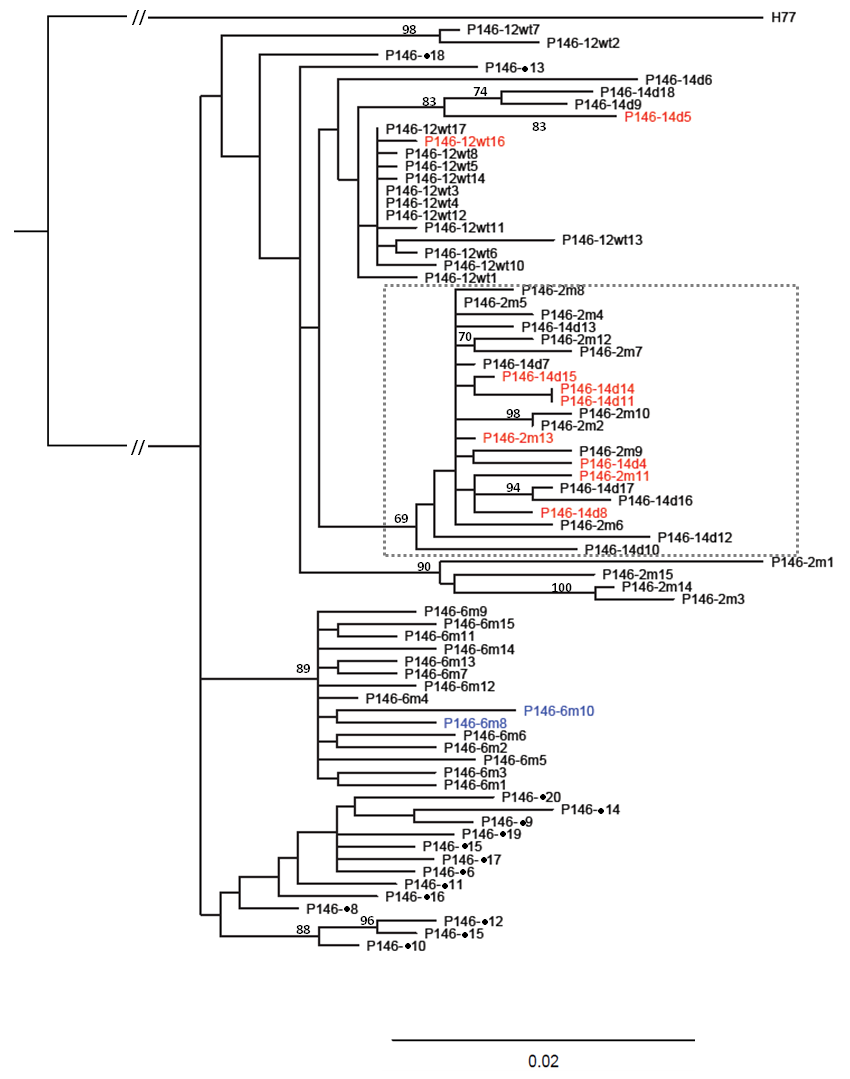

Supplement: Additional file 7 — Phylogenetic trees reconstructed from sequences obtained from patient P146 samples. Maximum likelihood tree reconstructed from full length NS5A region sequences obtained from samples of P146 (NR) plus reference sequence of genotype 1b HCV-J. The number of 1000 permuted trees supporting a clade indicated when that proportion was greater than 70%. The same quasispecies are colored in red. Sequence with nonsense mutation is colored in blue. The clade selected for selective pressure analysis is indicated by a gray line. [file 1471-2334-13-61-S7.tiff]

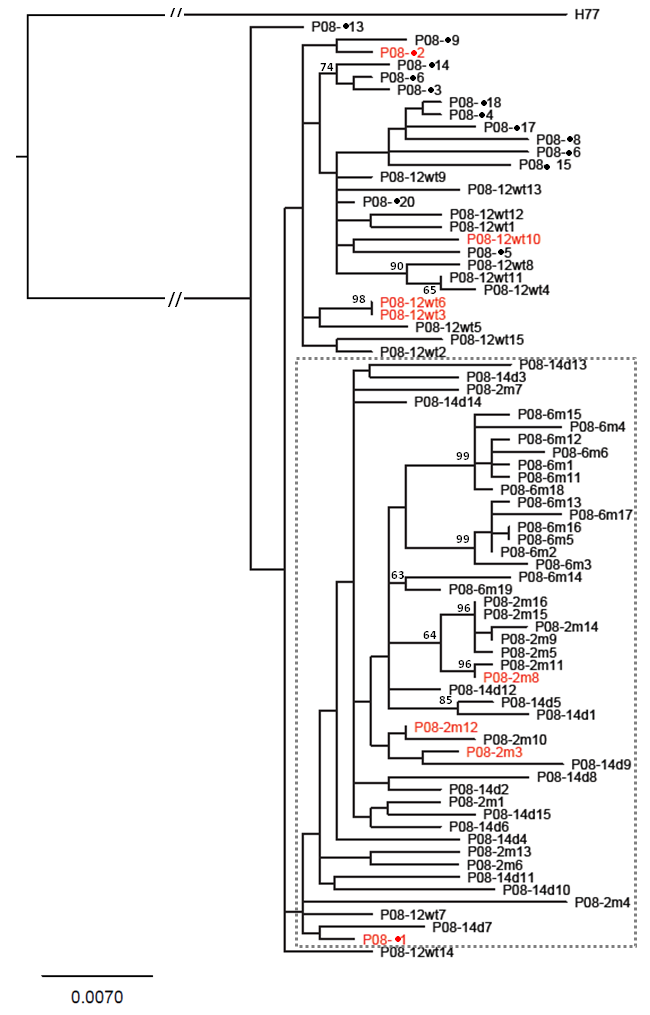

Supplement: Additional file 8 — Phylogenetic trees reconstructed from sequences obtained from patient P08 samples. Maximum likelihood tree reconstructed from full length NS5A region sequences obtained from samples of P08 (NR) plus reference sequence of genotype 1b HCV-J. The number of 1000 permuted trees supporting a clade indicated when that proportion was greater than 70%. The same quasispecies are colored in red. The clade selected for selective pressure analysis is indicated by a gray line. [file 1471-2334-13-61-S8.tiff]
